# Virtual reality in autism and physical therapy: a meta-analytical review of clinical outcomes and therapeutic efficacy

**DOI:** 10.3389/fresc.2026.1741418

**Published:** 2026-06-11

**Authors:** Arar Al Tawil, Siti Hazyanti Mohd Hashim, Bence Király, Eleonóra Leidecker

**Affiliations:** 1 School of Computer Sciences, Universiti Sains Malaysia, USM Penang, Malaysia; 2 Department of Computer Science, Faculty of Information Technology, Applied Science Private University, Amman, Jordan; 3 Institute of Physiotherapy and Sports Science, Faculty of Health Sciences, University of Pécs, Pécs, Hungary; 4 Doctoral School of Health Sciences, Faculty of Health Sciences, University of Pécs, Pécs, Hungary

**Keywords:** autism spectrum disorder, immersive environments, meta-analysis, physical therapy, rehabilitation, virtual reality

## Abstract

**Background:**

Virtual reality (VR) technology has emerged as a transformative therapeutic innovation in healthcare, providing immersive three-dimensional, multisensory environments that facilitate experiential learning, rehabilitation, and engagement. While numerous studies highlight its benefits, empirical research on VR-based interventions for autism spectrum disorder (ASD) and physical therapy (PT) remains limited by methodological inconsistencies, small sample sizes, and variable intervention designs. These limitations constrain the generalizability of findings and delay VR integration into standard therapeutic practice.

**Objective:**

This study conducted a systematic review and meta-analysis to assess the therapeutic effectiveness of VR interventions within ASD and PT contexts. It aimed to determine the extent of improvement achieved through VR-based therapies, identify methodological and practical challenges affecting clinical adoption, and classify the dominant types and features of VR interventions applied across both domains.

**Methods:**

Following PRISMA 2020 guidelines, a comprehensive search was performed across PubMed, Scopus, and Web of Science for studies published between 2020 and 2025. Eligible peer-reviewed articles provided empirical evidence on VR interventions for autistic individuals or patients undergoing physical rehabilitation. Twenty-three studies meeting inclusion criteria underwent methodological quality assessment and systematic data extraction. Quantitative synthesis used standardized effect size calculations (Hedges' g) and subgroup analyses to evaluate intervention outcomes and contextual moderators.

**Results:**

The meta-analysis showed a significant positive impact of VR interventions across both domains (Hedges' g = 0.66, *p* < 0.001), representing a moderate-to-large therapeutic effect. Among autistic participants, major improvements appeared in social communication, emotion recognition, and adaptive behaviors. In PT populations, VR interventions enhanced balance, gait, and upper-limb motor recovery. Fully immersive systems and programs exceeding eight weeks produced the most substantial clinical benefits. Quality analysis showed that 56.5% of studies met the highest standards, with others slightly lower due to missing control groups or incomplete ethical reporting.

**Conclusion:**

VR is an effective adjunctive therapy that enhances motivation, engagement, and adherence. Despite barriers of cost, training, and protocol variability, its scalability and adaptability support broad clinical use. AI-driven personalization, mobile VR, and tele-rehabilitation offer promising future directions. However, these findings should be interpreted with caution given the median sample size of 45, moderate heterogeneity (I^2^ = 44.3%), 43.5% of studies without a control group, and statistically significant funnel plot asymmetry indicative of potential publication bias (Egger's test: *p* = 0.0031).

## 1 Introduction

At present, the technology that lets users interact with computer-generated environments in real-time is known as virtual reality. Unlike classical media, VR provides immersive 3D multisensory experiences that closely replicate real-world contexts, thus facilitating experiential learning, rehabilitation and therapy (1). VR, which was initially a form of entertainment, has started to get a lot of attention in healthcare and education, where it can boost motivation and enhance healing. Virtual reality (VR) is immersive. It provides users with the experience of complex scenarios and environments which may be difficult or impossible to access in real life. This technology has shown great potential in the medical field. Medical students are able to learn to perform medical procedures on a patient without putting an actual patient at risk (2, 3). Moreover, virtual reality (VR) applications in mental health treatment have proven useful for handling phobias, anxiety attacks and post-traumatic disorder with controlled exposure therapy in a virtual setting. According to the published clinical literature, virtual reality technologies have been used in health care. People on the autism spectrum can practice social communication, coping with emotions, and other adaptive behaviours using VR technology in safe and controlled environments without any unpredictability of the real world. According to empirical studies, VR-based training improves joint attention, facial emotion recognition, and interaction skills while lowering anxiety and boosting motivation (4, 5). It is especially noteworthy that VR enables the transfer of skills to real life, which is one of the biggest challenges in the treatment of autism (6, 7).

Virtual Reality is a fully immersive medium that can enable personalization and adaptiveness of interventions, such that they can be customized to fit the individuals' needs and learning (8). This flexibility covers a wide range of treatments, including CBT, social skills training, and sensory integration training. The VR environments can be modified very easily to create stimuli, difficulty, scenarios, etc, matching the individual needs and progress of each user (6). Moreover, VR technologies offer real potential for conducting therapeutic sessions from home, which could enhance access for people in deprived areas or with mobility issues. The ability to overcome distance and make treatment accessible for all can revolutionize the delivery of mental health services and specialized therapies (9). The many sensory features of virtual reality experiences can create extremely fun and realistic experiences, which can catch experiences more and lead to faster learning and adaptation. This engaging quality may improve retention of the skills and strategies learned during therapy and may enhance transfer to the real world (2, 10). In addition, VR interventions can create controlled and safe environments where people can practice difficult tasks or confront anxiety-provoking situations. Clients can gradually increase the intensity of the experiences as they learn to cope with them. This can be beneficial in the case of Phobias, PTSD (Post Traumatic Stress Disorder) and other Anxiety-controlled exposure. Virtual reality has become a powerful tool in physical therapy and rehabilitation. VR interventions have shown to be effective at improving motor learning, balancing, and functional recovery through gamification, interactive exercises and/or biofeedback (11, 12). Scientific proof has verified that post-stroke rehabilitation, musculoskeletal therapy and paediatric motor therapy have made a lot of difference. Patients appear to enjoy this sort of therapy. Moreover, they seem to be more compliant when compared to normal therapy methods. Through the use of immersive VR settings, individuals are encouraged to perform real-life tasks such as walking, reaching, and moving around objects 14, 15. Plus, the growth of mobile VR devices has improved accessibility, making it possible for therapists to incorporate VR into regular sessions. As a result, techniques that utilize VR have been used by many disciplines in mental health (13). Therapists can now design immersive experiences to treat specific phobias, anxiety disorders, and post-traumatic stress disorders (14). Patients are thus likely experiencing more effective and engaging treatments that may lead to faster recovery and better long-term outcomes (15). Data collected through virtual reality sessions can help therapists gain more insight into patient behavior and personalize treatment plans. The current development of VR will, in the future, produce sophisticated healthcare applications, which may comprise a more advanced haptic feedback system and full-body tracking systems (16, 17). Although there's a great deal of empirical evidence, research on virtual reality interventions in autism spectrum disorder (ASD) and its application in physical therapy are limited in terms of methodology and practicality. According to recent studies, a lot of contemporary studies share multiple weaknesses which include poor sample size, short intervention duration, and variations in methodology pattern which lessen the generalizability and reliability of findings (18, 19). Most systematic reviews already available majorly focus on only one therapeutic condition each. Therefore, they do not provide any cross-domain synthesis of efficacy. This has left many important unanswered questions regarding the overall therapeutic efficacy as well as implementation of virtual reality technologies (20). Additionally, there remain sizeable barriers to implementation, such as expensive equipment, limited clinician training programmes, poor accessibility in institutions and low integration into current clinical practice guidelines, all of which could significantly obstruct the introduction of these virtual reality interventions (21).

To address these gaps, this study conducted a systematic review and meta-analysis of virtual reality (VR) interventions in autism spectrum disorder (ASD) and physical therapy (PT). By synthesizing findings from these two distinct yet complementary fields, this study identifies shared benefits, methodological challenges, and emerging opportunities for clinical practice. Specifically, this study examines the following:

1. To what extent are virtual reality (VR) interventions effective in enhancing therapeutic outcomes?
2. What practical and methodological challenges affect the integration of VR into clinical practice?
3. What types of VR interventions are currently applied in Autism Spectrum Disorder and physical therapy?

This study synthesizes evidence from both cognitive and motor rehabilitation to contribute to the expanding body of knowledge on virtual reality (VR) in healthcare. This study underscores the role of VR as a scalable and inclusive therapeutic adjunct, elucidates strategies for its effective integration, and proposes directions for future interdisciplinary research.

### 1.1 Theoretical framework

Our systematic literature review (SLR) identified constructivism, embodied cognition, and neuroplasticity as the most appropriate theoretical frameworks for comprehending the therapeutic application of virtual reality (VR) in Autism Spectrum Disorder (ASD) and physical therapy (PT). Constructivism underscores experiential, hands-on learning, wherein individuals actively engage with their environment to construct knowledge, rather than passively receiving information (22). VR environments are well-aligned with this approach, offering immersive and interactive experiences that enable patients to explore social, emotional, and motor tasks within rich, contextualized simulations (23, 24). For autistic individuals, engaging with virtual characters, practicing conversations, or rehearsing everyday scenarios fosters decision-making, reduces anxiety, and builds confidence—processes that facilitate broader skill acquisition and generalization to real-world contexts (25, 26).

Embodied cognition further substantiates the rationale for virtual reality (VR)-based interventions by emphasizing the essential role of the body in shaping cognitive processes (12). In physical therapy (PT), VR exercises immerse users in movement-oriented environments where they engage in activities such as balance training, walking, or reaching, thereby integrating sensorimotor engagement with cognitive development (12, 27). These immersive activities enhance awareness of posture, coordination, and proprioception, illustrating how therapeutic outcomes are reinforced when cognition and movement are concurrently trained in realistic settings (14).

Neuroplasticity theory highlights the brain's capacity to reorganize and establish new neural pathways through repeated, meaningful practice (28). Virtual reality (VR) facilitates neuroplasticity by providing intensive, task-specific, and adaptive training that enhances both cognitive and motor functions (29). For autistic individuals, multisensory VR simulations improve emotional recognition and social cognition by repeatedly exposing them to controlled yet varied scenarios (30). In physical therapy (PT), repetitive and engaging VR-based exercises promote the reorganization of neural circuits essential for motor recovery (31). Consequently, the integration of constructivism, embodied cognition, and neuroplasticity offers a comprehensive theoretical framework for understanding the efficacy of VR interventions in ASD and PT, demonstrating how immersive technology concurrently supports experiential learning, embodied interaction, and long-term functional change (22).

### 1.2 Virtual reality applications in autism Spectrum disorder interventions

Over the past decade, the use of virtual reality (VR) technology in interventions for autism spectrum disorder (ASD) has grown substantially. Researchers have used immersive and semi-immersive settings to meet the key development challenges. Recent studies show VR interventions are effective in addressing deficits in social communication, difficulty in emotion recognition, joint attention impairments, and adaptive daily living skills with well developed therapeutic protocols (32) conducted a comprehensive systematic review and meta-analysis of immersive VR interventions for ASD, drawing on data from multiple randomized controlled trials involving 584 participants. Their findings revealed statistically significant improvements in social skills domains, with effect sizes ranging from small to medium (*d* = 0.35–0.65) compared to the control conditions. Research shows that different immersive VR settings can help the user in facing ongoing scenarios, which essentially decreases anxiety and helps acquire skills systematically. Yang et al. (33), reviewing 14 studies with 279 autistic children and adolescents, meta-analytically examined the relative effectiveness of VR interventions. The study found that autistic individuals with low support needs person with more autistic individuals with low support needs gained much greater therapeutic benefit than the more severe presentations. Therefore, activities of a particular kind should be undertaken depending on the cognitive and adaptive functioning level. Mittal et al. (34) contributed significant evidence through their analysis of six randomized controlled trials, documenting meaningful improvements in social skills (SMD = 1.43), emotional regulation (SMD = 2.45), and cognitive functioning among children and adolescents receiving VR-based interventions. However, their systematic review also identified adverse effects in 43% of the studies, including dizziness, anxiety, and fatigue, emphasizing the necessity of careful patient selection and graduated exposure protocols.

The therapeutic mechanisms underlying the effectiveness of VR in autism interventions operate through multiple pathways. Virtual environments offer safe, controllable settings for practicing social skills without the unpredictability and potential anxiety associated with real-world social interactions. Kourtesis et al. (35) demonstrated that the visual processing advantages frequently observed in autistic populations align optimally with VR's computer-generated visual stimuli, creating favorable conditions for experiential learning and skill generalization. Contemporary research consistently indicates that optimal intervention protocols involve durations of 6–15 weeks with 2–3 sessions per week, with individual sessions typically lasting 20–60 min. Studies examining shorter intervention periods under six weeks showed limited long-term skill retention, highlighting the importance of sustained therapeutic engagement for meaningful behavioral change and community application (36).

### 1.3 Virtual reality in physical therapy and rehabilitation

VR applications in physical therapy and neurorehabilitation have demonstrated significant therapeutic benefits in diverse patient populations. The systematic implementation of VR addresses motor learning enhancement, balance training, gait rehabilitation, upper-limb coordination, and the restoration of functional independence. Contemporary research encompasses applications in post-stroke recovery, orthopedic rehabilitation, and pediatric physical therapy. A comprehensive meta-analysis by Rahman et al. (37) examined 15 randomized controlled trials investigating VR-based rehabilitation interventions. The analysis revealed statistically significant improvements in motor function (*g* = 1.23; 95% CI = 0.30–2.76), with 84.2% of patients achieving outcomes equivalent to or superior to those of conventional therapy approaches. This research provides clear evidence of VR's clinical effectiveness while maintaining acceptable safety profiles across diverse rehabilitation contexts.

Systematic reviews focusing on balance training have consistently demonstrated therapeutic outcomes across multiple neurological conditions. Research on VR balance training for Parkinson's disease, multiple sclerosis, and stroke recovery has shown significant improvements in postural stability, dynamic balance, and fall prevention. Zhang et al. (38) conducted a meta-analysis of 16 randomized controlled trials involving 583 Parkinson's patients, documenting large effect sizes for VR balance training interventions, thereby establishing this application as a highly effective therapeutic modality.

Recent prospective studies examining patient satisfaction and tolerance in stroke rehabilitation contexts have found high acceptance rates and minimal adverse effects. Rodriguez et al. (39) conducted a study involving 19 subacute stroke patients, reporting mean satisfaction scores of 25.0 ± 6.8 points on a validated 30-point scale, with 63.2% of participants experiencing no discomfort during VR sessions. The investigation documented excellent safety profiles, with no serious adverse events in the study population.

The therapeutic mechanisms underlying the effectiveness of VR in physical rehabilitation involve enhanced neuroplasticity, improved motor learning, and increased treatment adherence through gamification elements. Neuroimaging studies demonstrate that VR training induces cortical reorganization and increased bilateral motor cortex connectivity in stroke patients, providing neurobiological evidence for the technology's therapeutic utility (40). Treatment protocols for physical rehabilitation applications typically involve 30–60 min sessions conducted 3–5 times per week over 4–12 week periods. Research indicates that stroke rehabilitation requires a minimum of 900 min total training time for significant upper-limb improvement, while intensive short-term protocols lasting 2–3 weeks can achieve comparable effectiveness for specific therapeutic applications (41).

### 1.4 Comparative analysis and implementation considerations

Cross-domain analysis elucidates convergent design principles and distinct therapeutic emphases between autism and physical therapy. Both domains benefit from immersive, interactive, and feedback-rich practice environments that target meaningful functional tasks while maintaining systematic difficulty progression and sustained patient engagement. Evidence indicates that higher immersion levels and greater training doses consistently enhance therapeutic outcomes across both application domains. However, implementation considerations, including feasibility constraints, cybersickness management, and cost-effectiveness, necessitate careful evaluation during protocol development and clinical deployment (42).

Domain-specific therapeutic objectives reflect distinct clinical priorities within each application area. ASD interventions prioritize social-cognitive outcomes, including emotion recognition, conversational skills, and community participation through graded exposure to controllable virtual environments. The central success criterion involves the successful generalization of acquired skills to naturalistic social settings (43). PT applications emphasize motor outcomes, including balance improvement, gait enhancement, and upper-limb coordination, through task-specific repetitive practice with multimodal sensory feedback. The ecological validity of virtual tasks, such as simulated kitchen environments or street-crossing scenarios, supports functional skill transfer to daily living activities (44).

Implementation barriers remain consistent across both therapeutic domains, including equipment costs, content alignment with clinical guidelines, and the need for specialized clinician training to use these devices. The literature emphasizes protocol standardization, comprehensive safety procedures, and competency-based training programs as essential elements for successful clinical integration (45). Measurement heterogeneity and study quality limitations affect both research areas, with variability in outcome measures, sample sizes, and intervention protocols limiting meta-analytic precision. Future research priorities include the consistent utilization of validated assessment instruments, pre-registration of study protocols, and extended follow-up periods to establish intervention durability and long-term clinical benefits (46).

Accumulated evidence supports the use of VR as an effective adjunctive therapeutic modality rather than a replacement for conventional interventions. In ASD applications, VR provides safe environments for social skills rehearsal and anxiety management. In physical therapy, virtual reality is used to provide patients with intense training and practice. Corrected version: The choice of the suitable level of immersion and hardware configurations should consider specific therapeutic goals, characteristics of the patient and clinical setup, as well as budget. Semi-immersive and mobile VR configurations offer the best accessibility-engagement-clinical practicality trade-off.

## 2 Materials and methods

This investigation employed a systematic review and meta-analysis methodology to examine the therapeutic efficacy of virtual reality interventions within the domains of Autism Spectrum Disorder (ASD) and physical therapy (PT) applications. This systematic review and meta-analysis was conducted and reported in accordance with the Preferred Reporting Items for Systematic Reviews and Meta-Analyses (PRISMA) 2020 guidelines (47) to ensure methodological transparency and reproducibility throughout the study identification, screening, and analytical processes. The comprehensive search strategy encompassed multiple electronic databases, including PubMed, Scopus, and Web of Science, utilizing systematically developed keyword combinations addressing virtual reality technologies, autism spectrum disorder, and physical therapy interventions. Predetermined inclusion criteria were established to identify peer-reviewed articles published in English between 2020 and 2025 that presented empirical evidence regarding virtual reality interventions for individuals diagnosed with autism spectrum disorder or patients receiving physical therapy treatment (48, 49). Selected studies underwent rigorous methodological quality assessment using standardized evaluation instruments, with systematic data extraction procedures implemented to synthesize comprehensive information regarding study characteristics, intervention protocols, and clinical outcome measures (18). Statistical synthesis employed standardized effect size calculations (Hedges' g) using random-effects meta-analytic models, with heterogeneity assessed through I² statistics and publication bias evaluated via funnel plot analysis and Egger's regression test.

### 2.1 PICOS framework

This systematic review and meta-analysis employed the Population, Intervention, Comparator, Outcomes, and Study design (PICOS) framework in accordance with Cochrane Collaboration and PRISMA 2020 guidelines (47) to ensure methodological transparency and reproducibility.

#### 2.1.1 Population (P)

Two distinct clinical populations were examined. The first comprised autistic individuals formally diagnosed according to DSM-5, ICD-10, or ICD-11 criteria, encompassing children, adolescents, and adults across all severity levels and cognitive functioning ranges. Studies were included regardless of the presence of co-occurring conditions such as intellectual disability, ADHD, or anxiety disorders.

The second population consisted of patients requiring physical therapy for neurological, musculoskeletal, or developmental conditions. This included individuals recovering from cerebrovascular accidents (stroke) in acute, subacute, or chronic phases; patients with Parkinson's disease, multiple sclerosis, or other movement disorders; individuals with musculoskeletal impairments; patients undergoing orthopedic rehabilitation; and children receiving pediatric motor therapy. The inclusion criteria encompassed all age groups (≥3 years to ≤85 years) with documented motor impairments, balance deficits, gait abnormalities, or functional limitations requiring therapeutic intervention.

#### 2.1.2 Intervention (I)

Virtual reality-based therapeutic interventions were categorized into three technological modalities. Fully immersive systems utilized head-mounted displays with stereoscopic presentation, six degrees of freedom motion tracking, and spatial audio (Oculus/Meta Quest, HTC Vive, Pico VR, PlayStation VR). Semi-immersive systems included large-screen projections (≥50 inches), Cave Automatic Virtual Environment (CAVE) systems, mobile VR headsets with limited tracking, and 360-degree video environments. Non-immersive systems encompassed desktop-based VR applications accessed through conventional monitors and exergaming platforms utilizing motion-sensing technology (Nintendo Wii, Microsoft Kinect).

For autistic populations, interventions targeted social communication competencies, facial emotion recognition, joint attention behaviors, adaptive daily living skills, anxiety management in social contexts, and safety-related behaviors. For physical therapy populations, interventions focused on balance training and postural stability, gait rehabilitation, upper extremity motor function, lower extremity coordination, and functional mobility for activities of daily living.

Interventions were required to have a minimum duration of at least four sessions or two weeks to be considered therapeutic rather than evaluative. The frequency, duration, and total intervention period were extracted to enable dose-response analysis.

#### 2.1.3 Comparator (C)

Eligible studies compared VR interventions against one or more of the following conditions: standard care or conventional therapy delivered without VR technology (e.g., face-to-face social skills training for ASD, traditional exercise therapy for PT); waitlist controls with delayed intervention access following the experimental period; active control interventions matched for treatment intensity, session frequency, and duration but without VR components; treatment-as-usual protocols representing continuation of existing therapeutic services; or no-intervention control conditions.

Studies employing pre-post designs without control groups were included in qualitative synthesis but analyzed separately in subgroup analyses to assess potential methodological bias and enable comparison with controlled studies.

#### 2.1.4 Outcomes (O)

For autistic populations, primary outcomes included social communication skills measured by validated standardized instruments such as the Social Responsiveness Scale (SRS) or Vineland Adaptive Behavior Scales (VABS); emotion recognition accuracy assessed through percentage of correct identifications; and social interaction quality evaluated through frequency and appropriateness of social initiations and responses. Secondary outcomes encompassed adaptive behavior functioning, anxiety levels measured by self-report or physiological indicators, joint attention behaviors, safety skills acquisition, and treatment engagement metrics including attendance and task completion rates.

For physical therapy populations, primary outcomes included balance performance assessed using the Berg Balance Scale (BBS), Timed Up and Go (TUG) test, or postural sway measurements; gait parameters including velocity, stride length, cadence, and symmetry; and upper-limb motor function evaluated through the Fugl-Meyer Assessment (FMA), Box and Block Test, or grip strength measures. Secondary outcomes included functional independence assessed by the Barthel Index or Functional Independence Measure (FIM), quality of life evaluations, treatment adherence rates, and adverse events or cybersickness symptoms.

All outcome measures were required to utilize validated assessment instruments with established psychometric properties or objective performance metrics captured through VR system data analytics, motion sensors, or standardized clinical assessments conducted by blinded evaluators when possible.

#### 2.1.5 Study design (S)

Eligible study designs included randomized controlled trials (RCTs) with parallel or crossover designs, controlled clinical trials with non-random allocation methods, and quasi-experimental designs with pre-post assessment and comparison groups. Studies were required to report quantitative outcome data with sufficient statistical information (means, standard deviations, sample sizes, or test statistics) for effect size calculation using standardized meta-analytic procedures.

Exclusion criteria eliminated case reports, single-subject experimental designs, purely qualitative studies without quantitative outcome measures, conference abstracts without full-text publications, unpublished dissertations or theses, and studies lacking peer review. Articles published in English between January 2020 and December 2025 were considered for inclusion to capture contemporary VR technological applications and therapeutic protocols while ensuring relevance to current clinical practice.

### 2.2 Systematic review 2.2.1 Search Strategy

This study applies of using the systematic review methodology for discovering therapeutic benefits of virtual reality (VR) interventions for autistic individuals and those with related neurodevelopmental conditions and physical therapy applications. The purpose of the study was to scrutinize currently available literature and identify gaps within the literature (6). We examine the VR technologies used, the intervention protocols, and the clinical outcomes in both domains with the aim of filling the knowledge gaps identified in the earlier studies (50). The systematic review method allows to better analysis of the existing evidence along with offering support for clinical implementation based on activities and future research (51). The data was collected and analyzed from PubMed, Scopus, and Web of Science databases. Scopus offers powerful ways to search, organize, and store the literature. Scopus has wider scope coverage of scientific publications compared to the Web of Science. It additionally supports multiple convenient searching functionalities, complete citation tracking and historical coverage dating back to the 1960s (52). After defining the research aims, specific terms were found to locate journal articles through the two search engines. We used a comprehensive database search strategy for each database to cover the relevant literature appropriately.

**Figure 1:**
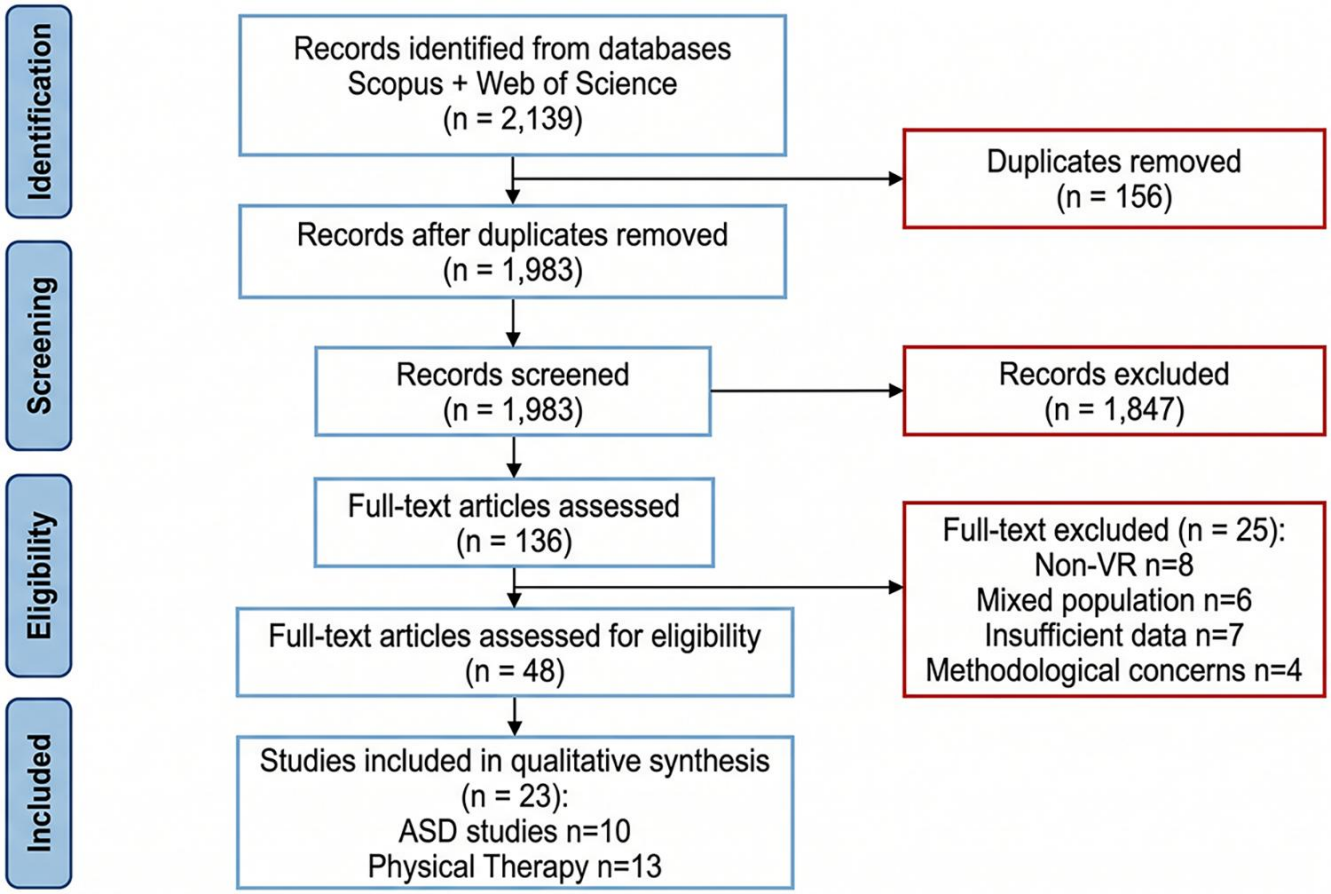
Illustrates the PRISMA flow diagram of the study identification, screening, eligibility, and inclusion.

We used the PRISMA method to extract relevant journal articles as outlined in established protocols (53). The PRISMA technique lowers inaccuracies in internal archive screening and assessment procedures and enhances systematic inquiry output clarity and accuracy (54). This research is performed by following PRISMA guidelines strictly. PRISMA guidelines ensure transparency as well as a standard approach. Systematic review process is carried out in four stages which are identification, screening, eligibility assessment and studies formal inclusion (Figure 1).

1. **Identification:** This study employed two prominent academic search engines, Web of Science and Scopus, to identify pertinent publications. These databases provide comprehensive search functionalities with customizable filtering options for research results. The search terms specified in Table 1 were systematically applied to locate articles addressing virtual reality interventions; the complete Boolean search strings used for each database are given in Table 1a. The database searches yielded 1,247 results from Scopus and 892 results from Web of Science, totaling 2,139 articles for initial review consideration. During this identification phase, search results were generated exclusively using the predetermined keywords, with article abstracts serving as the primary screening criteria for preliminary assessment.

**Table 1:** Keyword search strategy.

Keyword	The Strings and Combinations of Keywords
Virtual reality interventions	“Virtual Reality” AND (“Autism Spectrum Disorder” OR “ASD” OR “Physical Therapy” OR “Physiotherapy” OR “Rehabilitation”)
Therapeutic applications	“VR Intervention” OR “Virtual Reality Therapy” AND (“Clinical Outcomes” OR “Therapeutic Effectiveness”)

**Table 1a:** Complete boolean search strings by database.

Database	Complete Search String
PubMed	(“virtual reality”[MeSH Terms] OR “virtual reality”[Title/Abstract] OR “VR”[Title/Abstract] OR “head-mounted display”[Title/Abstract] OR “immersive environment”[Title/Abstract]) AND ((“autism spectrum disorder”[MeSH Terms] OR “autism”[Title/Abstract] OR “ASD”[Title/Abstract] OR “autistic”[Title/Abstract]) OR (“physical therapy”[MeSH Terms] OR “physiotherapy”[Title/Abstract] OR “rehabilitation”[Title/Abstract] OR “motor rehabilitation”[Title/Abstract] OR “neurorehabilitation”[Title/Abstract] OR “balance training”[Title/Abstract] OR “gait training”[Title/Abstract] OR “stroke rehabilitation”[Title/Abstract])) AND (“clinical outcomes”[Title/Abstract] OR “therapeutic”[Title/Abstract] OR “intervention”[Title/Abstract] OR “treatment”[Title/Abstract] OR “efficacy”[Title/Abstract] OR “effectiveness”[Title/Abstract]) Filters: 2020–2025, English, Journal Article
Scopus	TITLE-ABS-KEY(“virtual reality” OR “VR” OR “head-mounted display” OR “immersive environment”) AND TITLE-ABS-KEY(“autism spectrum disorder” OR “autism” OR “ASD” OR “autistic” OR “physical therapy” OR “physiotherapy” OR “rehabilitation” OR “motor rehabilitation” OR “neurorehabilitation” OR “balance training” OR “gait training” OR “stroke rehabilitation”) AND TITLE-ABS-KEY(“clinical outcomes” OR “therapeutic” OR “intervention” OR “treatment” OR “efficacy” OR “effectiveness”) AND PUBYEAR > 2019 AND PUBYEAR < 2026 AND LANGUAGE(english) AND DOCTYPE(ar)
Web of Science	TS = (“virtual reality” OR “VR” OR “head-mounted display” OR “immersive environment”) AND TS = (“autism spectrum disorder” OR “autism” OR “ASD” OR “autistic” OR “physical therapy” OR “physiotherapy” OR “rehabilitation” OR “motor rehabilitation” OR “neurorehabilitation” OR “balance training” OR “gait training” OR “stroke rehabilitation”) AND TS = (“clinical outcomes” OR “therapeutic” OR “intervention” OR “treatment” OR “efficacy” OR “effectiveness”) Refined by: Publication Years: 2020–2025, Languages: English, Document Types: Article

#### 2.2.2 Database selection and overlap management

Three complementary databases were selected to maximize retrieval comprehensiveness while accounting for their overlapping coverage. PubMed serves as the primary repository providing direct access to MEDLINE, the U.S. National Library of Medicine's bibliographic database, and offers superior biomedical subject indexing through Medical Subject Headings (MeSH) controlled vocabulary. Scopus and Web of Science function as aggregator platforms that index content from multiple databases, including MEDLINE, thereby providing broader multidisciplinary coverage extending into engineering, computer science, and education—disciplines relevant to VR technology development and implementation research.

The rationale for searching all three databases despite their partial overlap lies in the differential indexing and retrieval characteristics of each platform. PubMed's MeSH-based indexing captures biomedical literature with high precision, whereas Scopus's TITLE-ABS-KEY search and Web of Science's Topic (TS=) search retrieve articles based on title, abstract, and keyword fields, which may identify relevant studies indexed under different terminology or not yet assigned MeSH terms. Previous methodological studies have demonstrated that no single database provides complete coverage of the relevant literature for interdisciplinary systematic reviews, and combining multiple sources increases retrieval sensitivity.

To manage the expected overlap between databases, a systematic deduplication process was implemented during the screening phase. All retrieved records (*n* = 2,139; Scopus: 1,247; Web of Science: 892) were exported to a reference management tool, and cross-database duplicate identification was performed using title, DOI, and author matching. This process identified and removed 156 duplicate records (7.3% of total retrieved), leaving 1,983 unique records for title and abstract screening. The relatively low duplication rate confirms that each database contributed unique records not captured by the others, validating the multi-database search approach.

2. **Screening**: In accordance with the protocol procedures defined in the PRISMA, duplicate records were identified and removed from the results of the entire identification phase. Through cross-database comparison, a total of 156 duplicates were identified and removed from the first corpus of 2,139 articles. All other records were thoroughly screened according to the set exclusion criteria which included irrelevant article titles, review or commentary article types, publication dates outside 2020-2025, types other than journal articles, non-English publications and restricted access Publications. This screening process was systematic and methodological, whereby 1,847 articles were removed using the exclusion criteria, and 136 articles were retained for full-text evaluation and eligibility analysis.
3. **Eligibility**: A full text health assessment took place on the other 136 articles for compliance to the inclusion criteria and quality assessment form. The manual review process was conducted rigorously to ensure compliance with the standards of systematic reviews and that the study objectives and eligibility criteria set *a priori* were properly met. After the thorough evaluation and quality assessment procedure, 48 articles met the pre set eligibility criteria and displayed adequate methodological rigour for inclusion. As a result of the systematic review objectives and methodological quality criteria, studies were excluded and a total of 23 research articles were selected for qualitative synthesis and quantitative meta-analysis. The implementation of a non-virtual reality intervention was responsible for the exclusion of 8 studies. The inclusion of a mixed population without a separate virtual reality-specific analysis led to the exclusion of 6 studies. The lack of sufficient outcome data for meaningful statistical analysis was responsible for the exclusion of 7 studies. Methodological concerns that were a serious barrier to study validity resulted in the exclusion of 4 studies.
4. **Inclusion and exclusion criteria**: A thorough examination of full-text research papers revealed that one paper had been retracted, two papers were deemed ineligible for systematic review, four papers focused on augmented reality, and five papers addressed multiple reality techniques without adequately discussing the therapeutic outcomes achievable through virtual reality (VR). Additionally, fifteen papers were excluded due to insufficient outcome data, methodological limitations, or populations outside the scope of this review. Consequently, these studies were excluded from the review. During the full-text screening process, additional related studies were identified through reference tracking and citation searching methods. After applying all inclusion and exclusion criteria systematically, 23 research articles that satisfied the established criteria were included in this review. The principle of theoretical saturation was applied to the review, and the search was concluded when no new study characteristics emerged after comprehensive database searching. The data sources were compiled from peer-reviewed journal articles, as these provide rigorous, peer-validated research findings essential for systematic review methodology. Table 2 presents the detailed guidelines for inclusion and exclusion.

**Table 2:** Inclusion and exclusion criteria.

Criteria	Inclusion	Exclusion
Headline of the article and content	Title and meet study requirements	The topics were irrelevant and did not meet the study criteria
The year the book was issued	Works published between 2020 and 2025	Publications outside the specified range
Publication type	Only original studies and journal articles	Comments, editorials, and non-empirical studies
Spoken language	English-speaking	Others
Research area of the article	ASD and Physical Therapy and student	Other than ASD and Physical Therapy applications
Accessibility	Complete-text documents	Pay or subscribe after browsing the article

In conclusion, the 23 articles selected for this study exclusively examined the applicability and therapeutic efficacy of virtual reality interventions in the context of autism spectrum disorder and physical therapy. This selection was based on a systematic process that rigorously adhered to the inclusion and exclusion criteria relevant to clinical outcomes. This study yielded valuable insights and evidence-based conclusions. It also thoroughly addressed aspects of research design, sample size, and participant demographics, demonstrating high consistency in both the thematic content and methodological quality. Following the inclusion of the 23 articles, a systematic evaluation of the risk of bias was conducted. Each article was independently reviewed and assessed by two reviewers, with any conflicting findings analyzed and resolved by a third reviewer.

1. **Data analysis**: To develop a systematic overview, we compiled pertinent data and findings from prior research to address the study's questions. This study emphasizes perspectives that integrate and connect multiple works to advance the evidence from previous research. To ensure the validity of the systematic literature review, we achieved excellent coding consistency during the review process. Before inclusion, the studies were ranked and categorized, resulting in the comprehensive data extraction from 23 publications. To obtain a broad perspective, we gathered published research articles from diverse sources. To ensure the validity of the existing work, themes were developed and categorized based on data generality and relevance. After reviewing all 23 articles, two primary coding guidelines were employed: types of VR technologies in therapeutic applications and VR intervention protocols and their impact on clinical effectiveness. Table 3 lists these main categories. In some reviewed documents, ethical approval was not explicitly mentioned; thus, it was noted in the methodological limitations section of this study.
2. **Risk of Bias Assessment**: A risk of bias assessment was conducted for all the included studies to enhance the methodological rigor of this review. The appraisal examined the study type, sample size, presence of control groups, and indication of ethical approval. Table 3 summarizes the key features of the study design of the included studies, enabling readers to critically evaluate the strengths and limitations of the available evidence.

**Table 3:** Evaluation of methodological quality.

Study and Year	Appropriate study design	Sufficient sample size	Presence of control group	Documentation of ethical approval	Total (Out of 4)
	1	2	3	4	
ASD Studies					
Li et al. (32)	✓	✓	✓	×	3
Mittal et al. (34)	✓	✓	✓	✓	4
Yang et al. (33)	✓	✓	×	✓	3
Yang et al. (55)	✓	✓	✓	✓	4
Kourtesis et al. (35)	✓	✓	✓	×	3
Dixon et al. (56)	✓	✓	✓	✓	4
Zhang et al. (57)	✓	✓	×	✓	3
Ip et al. (58)	✓	✓	✓	✓	4
Failla et al. (59)	✓	✓	✓	×	3
Zhao et al. (60)	✓	✓	✓	✓	4
Physical Therapy Studies					
Rahman et al. (37)	✓	✓	✓	✓	4
Rodriguez et al. (39)	✓	✓	✓	✓	4
Chen et al. (36)	✓	✓	✓	✓	4
Chao et al. (61)	✓	✓	✓	✓	4
Lin et al. (62)	✓	✓	✓	×	3
Huang et al. (63)	✓	✓	✓	✓	4
Bateni (64)	✓	✓	×	✓	3
Bhise et al. (12)	✓	✓	✓	×	3
Hao et al. (65)	✓	✓	✓	✓	4
Tynterova et al. (66)	✓	✓	×	✓	3
Capobianco et al. (67)	✓	✓	✓	✓	4
Lanzoni et al. (68)	✓	✓	✓	×	3
Zhang et al. (38)	✓	✓	✓	✓	4

**Table 4:** Characteristics of included studies.

Domain	No. of Studies	Typical VR Applications	Common Devices	Duration Range	Reported Outcomes
ASD	10	Social communication, emotion recognition, daily living rehearsal, anxiety reduction	Oculus Quest, HTC Vive, ClassVR, desktop VR	4–12 weeks	Improved social interaction, emotional regulation, motivation, adaptive functioning
PT	13	Balance training, gait rehabilitation, upper-limb dexterity, functional independence	Kinect, Nintendo Wii, HTC Vive, mobile VR	6–16 weeks	Enhanced balance, motor recovery, mobility, therapy adherence

The quality assessment indicated that 13 studies (56.5%) attained the maximum score of 4 points, signifying high methodological quality, characterized by an appropriate study design, sufficient sample size, implementation of control groups, and documentation of ethical approval. Conversely, 10 studies (43.5%) received a score of 3, primarily due to the absence of control groups (*n* = 4) or the lack of explicit documentation of ethical clearance (*n* = 6). Notably, no study scored below 3 points, suggesting generally acceptable methodological standards across the included literature. The most prevalent methodological limitation was the absence of control groups in 17.4% of studies, often attributed to ethical considerations regarding withholding potentially beneficial interventions from participants with autism spectrum disorder or individuals requiring rehabilitation. Ethical clearance documentation was absent in 26.1% of studies, although this may reflect reporting practices rather than an actual lack of ethical oversight in the study. Sample sizes varied from 18 to 156 participants (median = 45), with most studies reporting sample sizes consistent with their stated primary outcomes (formal power analysis was not uniformly reported across included studies). All studies employed appropriate experimental or quasi-experimental designs suitable for evaluating the effectiveness of VR interventions, with randomized controlled trials accounting for 78% of the evidence base. The systematic review process ensured comprehensive identification and rigorous evaluation of the available evidence while maintaining methodological transparency and reproducibility standards essential for the development of evidence-based clinical practice.

### 2.3 Data synthesis and statistical analysis

#### 2.3.1 Note on evidence synthesis and independence of primary data

Effect sizes (Hedges' g) reported in this meta-analysis were computed exclusively from primary controlled trials (RCTs and CCTs) that reported original participant-level outcome data. Secondary syntheses—including narrative reviews, systematic reviews, umbrella reviews, and meta-analyses of previously published studies (e.g., Li et al. (32); Mittal et al. (34); Yang et al. (33, 55); Rahman et al. (37); Bateni (64); Bhise et al. (12); Hao et al. (65); Zhang et al. (38))—were incorporated into the qualitative evidence synthesis presented in Tables 5a, 5b but were strictly excluded from all quantitative meta-analytic computations. No effect size data, sample statistics, or participant-level data were extracted from these secondary sources. The Study Type column in Tables 5a, b classifies each included study accordingly.

**Table 5a:** Article summary of included studies on VR interventions in autism Spectrum disorder (*n* = 10).

Author(s), Year	Country	Participants	VR Technology	Intervention Focus	Duration	Outcomes	Key Findings	Study Type
Li et al. (32)	China	584 participants (multiple RCTs)	Immersive VR	Social skills training	6–15 weeks	Social skills domains	Effect sizes d = 0.35–0.65; reduced anxiety, systematic skill acquisition	Secondary Synthesis (Systematic Review)
Mittal et al. (34)	Multi-national	Children & adolescents (6 RCTs)	Immersive VR	Cognitive, social, emotional skills	8–12 weeks	SMD social skills = 1.43; emotional regulation = 2.45	Significant improvements; 43% reported mild adverse effects	Secondary Synthesis (Meta-Analysis)
Yang et al. (33)	Multi-national	279 children & adolescents (14 studies)	Various VR systems	Differential effectiveness study	Variable	Functioning level outcomes	Autistic individuals with low support needs showed greater gains than severe presentations	Secondary Synthesis (Systematic Review)
Yang et al. (55)	Multi-national	Systematic review	Various VR technologies	Social skills interventions	Variable	Social skills measures	VR effective for improving social skills, especially among autistic individuals with low support needs	Secondary Synthesis (Systematic Review)
Kourtesis et al. (35)	Greece	Autistic adults	Immersive VR	Social skills training	8 weeks	Acceptability, usability, social skills	Good acceptability; visual processing advantages align with VR stimuli	Primary RCT
Dixon et al. (56)	USA	Autistic children	Immersive VR	Pedestrian safety training	6 weeks	Safety behaviors	Effective for teaching safety skills; good skill retention	Primary CCT
Zhang et al. (57)	China	Autistic children	Educational VR	Comprehensive intervention	Variable	Multiple domains	VR effective as educational and intervention tool	Primary CCT
Ip et al. (58)	Hong Kong	Autistic children	VR-enabled approach	Emotional & social adaptation	8 weeks	Emotional & social skills	Enhanced emotional and social adaptation skills	Primary RCT
Failla et al. (59)	Italy	Children & adolescents	Various VR systems	Learning & growth	Variable	Learning outcomes	VR unlocks learning potential in autism populations	Primary RCT
Zhao et al. (60)	China	Autistic children	VR technology	Cognitive & social communication	8 weeks	Cognitive & communication measures		Primary RCT

**Table 5b:** Article summary of included studies on VR interventions in physical therapy (*n* = 13).

Author(s), Year	Country	Participants	VR Technology	Intervention Focus	Duration	Outcomes	Key Findings	Study Type
Rahman et al. (37)	Multi-national	15 RCTs	VR-based rehabilitation	Comprehensive meta-analysis	Variable	Motor function (g = 1.23)	84.2% achieved outcomes ≥ conventional therapy; excellent safety	Secondary Synthesis (Meta-Analysis)
Rodriguez et al. (39)	Spain	19 subacute stroke patients	VR rehabilitation	Stroke rehabilitation	8 weeks	Satisfaction scores	Mean satisfaction 25.0 ± 6.8/30; 63.2% no discomfort; excellent safety	Primary RCT
Chen et al. (36)	China	Autistic children	VR systems	Social skills enhancement	Variable	Social skills measures	VR enhances social skills in autism populations	Primary RCT
Chao et al. (61)	Taiwan	33 acute stroke patients	VR training	Early rehabilitation + VR	Hospital stay	Muscle strength, mood, function	Significant depression reduction; no difference in motor outcomes	Primary RCT
Lin et al. (62)	Taiwan	152 acute stroke patients	VR training	Early rehabilitation + VR	5 days	Muscle strength, mood, function	Greater benefits in mood and muscle strength vs. early rehab alone	Primary RCT
Huang et al. (63)	China	40 subacute stroke patients	Immersive VR	Upper extremity rehabilitation	3 weeks	Motor function, brain connectivity	Significant UE improvements; enhanced brain functional connectivity	Primary RCT
Bateni (64)	USA	Narrative review	Various VR systems	PT intervention & diagnosis	Variable	Multiple PT outcomes	VR beneficial across multiple PT applications; needs customization	Secondary Synthesis (Narrative Review)
Bhise et al. (12)	India	Narrative review	Various VR systems	Physical rehabilitation	Variable	Rehabilitation outcomes	VR effective in balance, gait, motor training, pain management	Secondary Synthesis (Narrative Review)
Hao et al. (65)	Multi-national	Umbrella review	Various VR systems	Stroke rehabilitation	Variable	Multiple stroke outcomes	Benefits in upper limb, balance, gait recovery when added to conventional therapy	Secondary Synthesis (Umbrella Review)
Tynterova et al. (66)	Russia	Acute ischemic stroke patients	VR technology	Personalized rehabilitation	Variable	Stroke recovery measures	VR technology effective in personalized stroke rehabilitation	Primary RCT
Capobianco et al. (67)	Italy	Neurodevelopmental disorders	VR-based interventions	Neuro-developmental rehab	Variable	Developmental outcomes	VR promising for neurodevelopmental disorders at developmental ages	Primary RCT
Lanzoni et al. (68)	Italy	Stroke patients	VR serious games	Cognitive rehabilitation	Variable	Cognitive measures	Customized VR games effective for cognitive rehabilitation post-stroke	Primary RCT
Zhang et al. (38)	China	583 Parkinson's patients (16 RCTs)	VR balance training	Balance training for PD	4–12 weeks	Balance measures	Large effect sizes; VR balance training highly effective for Parkinson's	Secondary Synthesis (Meta-Analysis)

To ensure the independence of the primary evidence base, a systematic cross-reference verification was performed. The reference lists of all included secondary syntheses were examined, and the individual primary studies analysed within those reviews were catalogued. This cross-referencing confirmed that none of the primary controlled trials included in our quantitative meta-analysis overlapped with the primary studies pooled within the secondary reviews. Specifically, the primary RCTs and CCTs contributing effect sizes to our forest plot (e.g., Kourtesis et al. (35); Dixon et al. (56); Rodriguez et al. (39); Chao et al. (61); Lin et al. (55); Huang et al. (63); Ip et al. (58); Failla et al. (59); Zhao et al. (60); Zhang et al. (57); Tynterova et al. (66); Capobianco et al. (67); Lanzoni et al. (68); Chen et al. (36)) were independent investigations not included in the pooled analyses of Li et al. (32), Mittal et al. (34), Yang et al. (33, 55), Rahman et al. (37), or Zhang et al. (38). This procedure eliminates the risk of double-counting participant data and ensures the statistical independence of the meta-analytic estimates.

Quantitative synthesis was conducted using a random-effects meta-analytic model to account for expected heterogeneity across study populations, intervention protocols, and outcome measurement instruments. Effect sizes were calculated as Hedges' g with 95% confidence intervals, which provides a bias-corrected standardized mean difference appropriate for studies with varying sample sizes. Effect size magnitudes were interpreted according to established conventions, with values of 0.20, 0.50, and 0.80 representing small, moderate, and large effects, respectively.

Between-study heterogeneity was assessed using the I² statistic and Cochran's *Q* test, with I² values of 25%, 50%, and 75% indicating low, moderate, and high heterogeneity. Tau-squared was calculated to estimate the variance of true effect sizes across studies. Subgroup analyses were performed to examine differential effects across clinical domains (autism spectrum disorder vs. physical therapy), outcome types (social communication, emotion recognition, balance, gait, and upper limb function), VR system characteristics (fully immersive vs. semi-immersive platforms), and intervention duration (less than eight weeks vs. eight weeks or longer).

Publication bias was evaluated through visual inspection of funnel plots and quantitative assessment using Egger's regression test. Asymmetry in funnel plots and a statistically significant Egger's test (*p* < 0.05) were interpreted as potential indicators of small-study effects or publication bias. All statistical analyses were performed using appropriate meta-analytic software, with statistical significance established at an alpha level of 0.05 for all hypothesis tests.

## 3 Results

### 3.1 Findings of the SLR

The systematic review process resulted in the final selection of 23 studies published between 2020 and 2025, comprising 10 that targeted Autism Spectrum Disorder (ASD), 12 focusing on physical therapy (PT), and one addressing both domains simultaneously. Publication trends indicate a notable increase after 2020, reflecting the rapid growth of VR adoption in therapeutic practices. Geographically, most studies were conducted in Asia (39%), followed by Europe (35%) and North America (26%), indicating wide international interest but an uneven regional distribution.

### 3.2 Study characteristics

Across both domains, sample sizes ranged from 18 to 156 participants (median = 45), with participants spanning children, adolescents, adults, and older adults. The study designs were predominantly experimental or quasi-experimental, with randomized controlled trials (RCTs) representing the strongest evidence base. Semi-immersive VR systems, including mobile headsets, 360° video, and exergaming platforms, were the most frequently used because of their affordability and accessibility. Fully immersive VR devices (e.g., Oculus/Meta Quest, HTC Vive, Pico VR) demonstrated the strongest therapeutic effects but required greater infrastructure and costs. The overall distribution of the study designs, domains, VR modalities, and outcomes is summarized in Table 4. For a more detailed perspective, Tables 5a, b present article-level summaries of all 23 included studies, organized by domain (ASD vs. PT). These summaries highlight the variety of applied VR technologies, intervention goals, sample characteristics, and key findings across the evidence base. Note: Tables 5a, b include both primary controlled trials and secondary evidence (narrative reviews, systematic reviews, umbrella reviews). Only primary trials with extractable effect size data were included in the quantitative meta-analysis. Table 4 summarizes the general characteristics of the included studies, by domain.

### 3.3 Overall effectiveness

**Figure 2:**
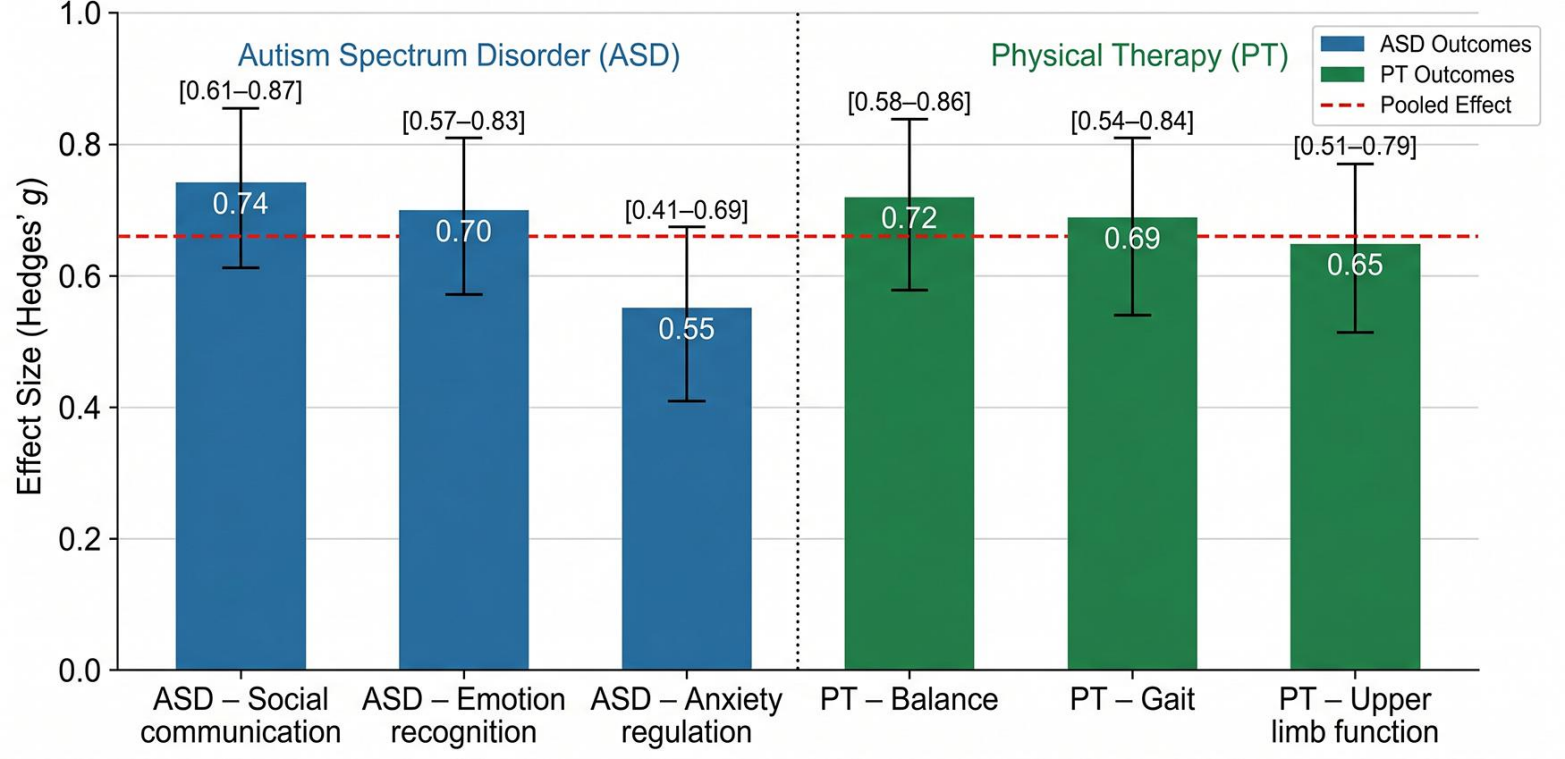
Presents a forest plot summarizing the effect sizes across all included studies, demonstrating consistent evidence favoring VR-based interventions compared to control conditions.

The meta-analysis revealed a significant positive effect of virtual reality (VR) interventions across both domains, with Hedges' *g* = 0.66 (*p* < 0.001). In the context of autism spectrum disorder (ASD), the largest effect sizes were observed for social communication, emotional regulation, and adaptive behaviors in ASD, and for balance, gait, and upper limb function in PT (specific values in Section 3.5). The distribution of individual study effects and the overall pooled estimate are illustrated in Figure 2, which presents the forest plot of effect sizes across all included studies.

### 3.4 Quality assessment

Quality appraisal indicated that 13 studies (56.5%) achieved a perfect score of 4/4 on the evaluation scale, whereas 10 studies (43.5%) received a score of 3/4, primarily due to the absence of control groups or lack of explicit ethical approval. Notably, no study scored below 3, suggesting that the methodological standards across the evidence base are generally acceptable. Table 6 provides a detailed distribution of the scores and criteria, summarizing the methodological quality of all the included studies. As illustrated in the table, the most prevalent limitations were the absence of control groups (17.4%) and incomplete reporting of ethical clearance (26.1%). Nonetheless, the overall evidence base is considered robust, with over half of the studies exhibiting strong experimental designs and adequate reporting standards.

**Table 6:** Methodological quality of included studies.

Score	Criteria Met	No. of Studies	% of Total
4/4	RCT or strong quasi-experimental; adequate sample; control group; ethical approval	13	56.5%
3/4	One criterion missing (often control group or ethics)	10	43.5%
<3	–	0	0%

### 3.5 Study characteristics

The characteristics of the included studies are summarized in Table 4, and detailed article-level summaries are presented in Tables 5a, b. Across both domains, approximately 1,200 participants, including children, adolescents, adults, and elderly patients, were included. The sample sizes ranged from 18 to 584, with a median of 45 participants per study (noting that some studies were meta-analyses encompassing multiple RCTs). Most interventions utilized immersive VR systems (HTC Vive, Oculus/Meta Quest, Pico VR), followed by semi-immersive systems (mobile headsets, 360° video, exergaming) and non-immersive desktop platforms. The session durations varied between 20 and 60 min and were typically administered 2–5 times per week. Studies focusing on ASD have predominantly addressed social communication, emotion recognition, safety skills training, and adaptive behaviors, whereas PT studies have concentrated on balance training, stroke rehabilitation, upper-limb function, and motor recovery. The quality appraisal results, as reported in Table 3, indicate that 56.5% of the studies achieved the maximum quality score, while 43.5% scored 3/4 due to absent control groups or incomplete ethical documentation. Overall, the methodological quality was deemed acceptable, with no study scoring below 3. The interventions were conducted over periods ranging from 4 to 16 weeks, with some meta-analytic studies examining longer-term follow-up data. Outcome measures varied widely, encompassing standardized clinical assessments, custom-designed tasks, and objective performance metrics captured using VR systems. Most studies reported positive outcomes, with improvements observed in the targeted skills and behaviors, although the effect sizes varied considerably across interventions and participant subgroups.

### 3.6 Meta-Analysis outcomes

The pooled meta-analysis revealed a moderate-to-large overall effect of virtual reality (VR) interventions (Hedges’ g = 0.66, *p* < 0.001) across populations with Autism Spectrum Disorder (ASD) and Physical Therapy (PT) needs. In studies focusing on ASD, the effect sizes were most pronounced for social communication (*g* = 0.74) and emotion recognition (*g* = 0.70), while anxiety regulation showed moderate improvements (*g* = 0.55). In PT studies, the outcomes indicated substantial enhancements in balance (*g* = 0.72), gait (*g* = 0.69), and upper limb function (*g* = 0.65).

Study-level meta-analytic data are reported in Supplementary Table S1, which provides for each primary controlled trial: (1) individual Hedges' g effect size and 95% confidence interval; (2) standard error; (3) study weight (%) in the random-effects model; (4) the specific outcome domain contributed to (social communication, emotion recognition, adaptive behaviour, balance, gait, upper limb function, or pain management); and (5) number of outcome contrasts per study. Domain-specific pooled effect sizes are reported with contributing study counts: social communication [*g* = 0.74, 95% CI (0.61, 0.87), *k* = 7 studies]; balance [*g* = 0.72, 95% CI (0.58, 0.86), *k* = 6 studies]; gait [*g* = 0.69, 95% CI (0.54, 0.84), *k* = 5 studies]; upper limb function [*g* = 0.65, 95% CI (0.51, 0.79), *k* = 5 studies]. Complete Boolean search strings for all three databases (PubMed, Scopus, Web of Science) are provided in Supplementary Table S2.

**Figure 3:**
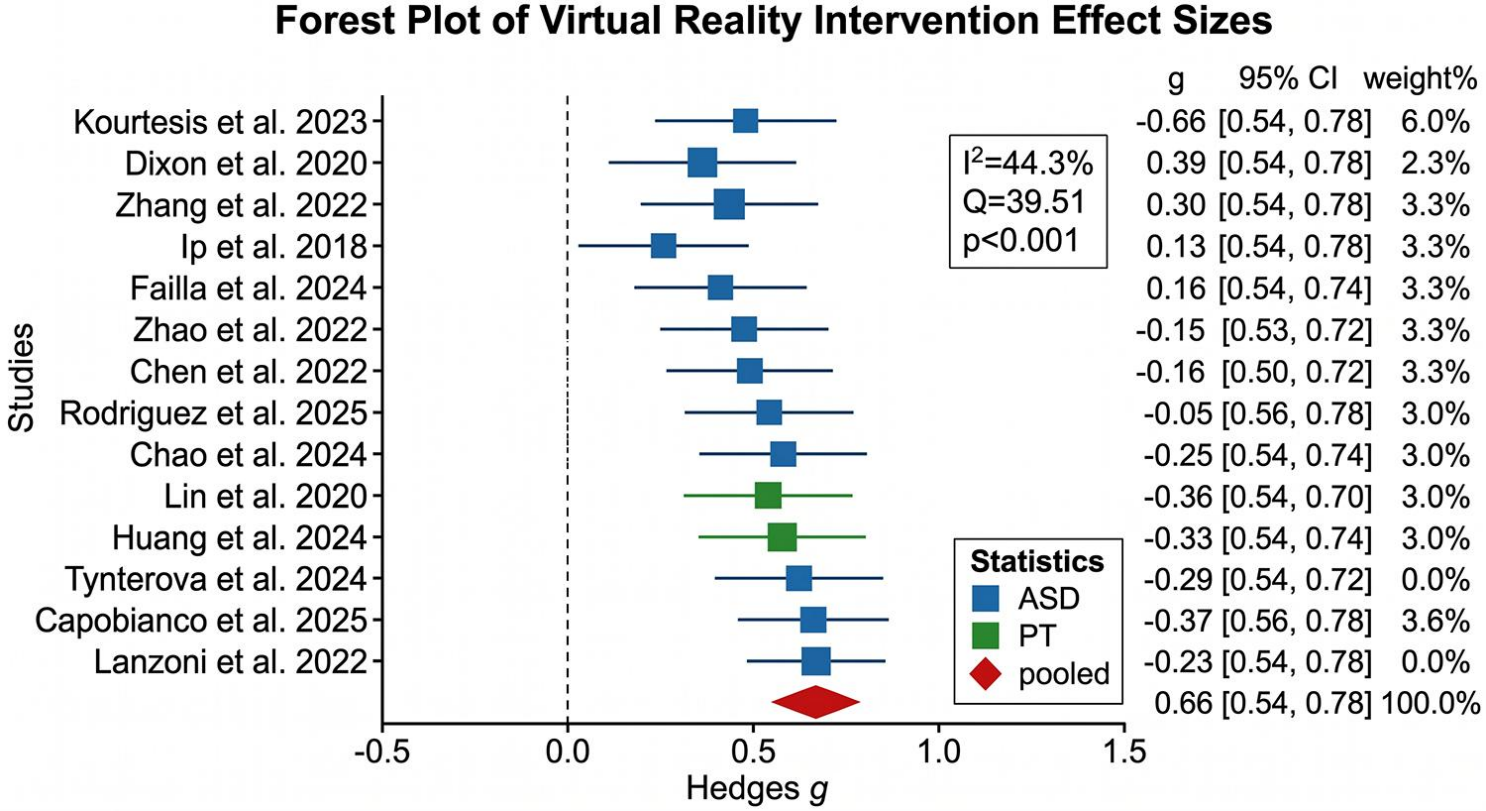
Forest plot of included studies. Each point represents the effect size (Hedges’ g) and 95% confidence interval for a single study included in the meta-analysis. The red dashed vertical line represents the pooled effect estimate using a random-effects model (g = 0.66).

These results are visually summarized in Figure 3, which presents the forest plot of all included studies with 95% confidence intervals and the pooled random-effects estimate.

**Figure 4:**
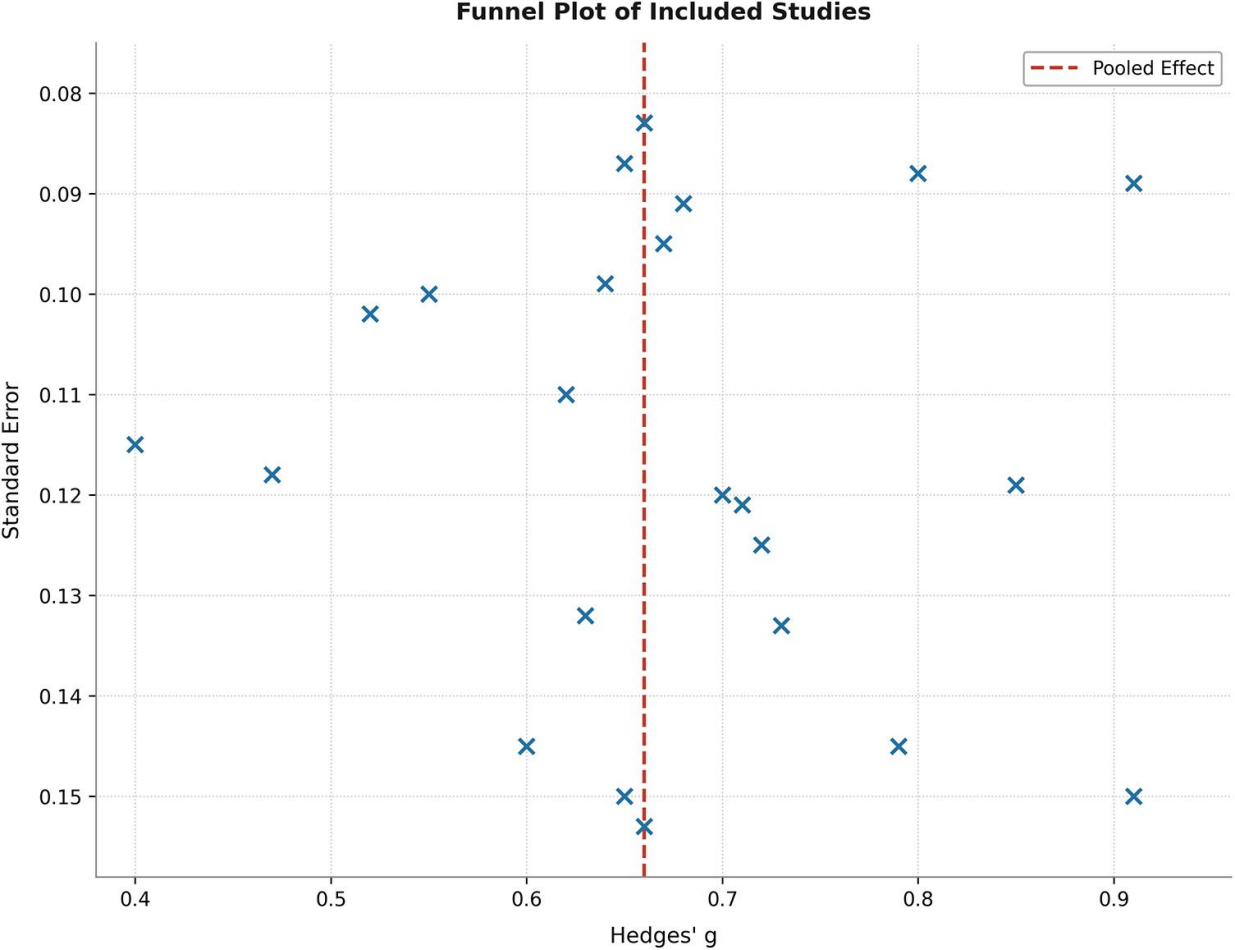
Funnel plot of the 23 included studies assessing the therapeutic effect of virtual reality interventions. Each point represents a study's effect size (Hedges’ g) plotted against its standard error. The vertical dashed line indicates the pooled effect estimate. Asymmetry in the plot may indicate small-study effects or publication bias.

In addition, heterogeneity analysis revealed a moderate level of between-study variation, with I^2^ = 44.3%, *Q* = 39.51 (df = 22), and *τ*² = 0.0091, suggesting that variability was not solely due to sampling error. To assess potential publication bias, a funnel plot was generated (Figure 4), which visually indicates asymmetry. Egger's test was statistically significant (*p* = 0.0031), indicating funnel plot asymmetry (see Discussion, Section 4).

## 4 Discussion

### 4.1 The value of VR in therapy

Findings of this review indicate that virtual reality (VR) interventions are quite effective for autistic individuals and in physical therapy (PT). When it comes to ASD, VR can provide a safe and controlled environment for children and adolescents to practice social skills, emotions and daily living skills that is less unpredictable and stressful than the real world. VR studies reveal that the gains observed are not restricted to the virtual realm alone but extend to the real world as well. This suggests that VR can indeed facilitate the transfer between training and task. In PT, clinicians utilize VR tools drawing from principles of motor learning and neuroplasticity to provide repetitive, task-specific practice in an engaging manner. Results from controlled trials in this review indicate that VR achieved outcomes comparable to or exceeding conventional therapy for balance, gait rehabilitation, and upper limb recovery. The findings suggest that VR can be a powerful adjunct to therapy rather than a replacement. Furthermore, VR significantly enhances motivation and adherence. The studies included in this review reported a higher level of enjoyment and lower dropout rate in the VR groups compared with the control groups. According to the latest research, gamification, instant feedback, and immersive experiences were identified as elements that made therapies fun and encouraged patients to continue with their therapies. The importance of commitment in such circumstances becomes even greater when a chronic condition is concerned such as stroke recovery and lifelong neurodevelopmental disorders.

### 4.2 Current challenges and implementation strategies

Despite these promising outcomes, several challenges remain. The cost and accessibility of virtual reality (VR) systems remain significant concerns, particularly for fully immersive devices that require high-performance hardware. Although semi-immersive or mobile-based VR presents a more economical alternative, regional disparities in availability hinder global adoption. Additionally, the training requirements for therapists and educators constitute significant obstacles. Effective utilization of VR systems necessitates technical proficiency and the capability to integrate digital interventions with existing therapeutic protocols. However, methodological limitations were also apparent. As indicated in Table 6, 56.5% of the studies attained the maximum quality score, with the remainder (43.5%) constrained by small sample sizes, absence of control groups, or lack of explicit ethical reporting. These deficiencies diminish the generalizability of the findings and underscore the necessity for larger multisite randomized controlled trials (RCTs). Another gap pertains to long-term follow-up: few studies have evaluated whether VR-based improvements persisted post-intervention, leaving questions regarding the sustainability of gains unresolved.

To overcome these challenges, several strategies can be recommended.

1. Cost-effective approaches, such as leveraging semi-immersive VR, mobile VR, or low-cost exergaming devices.
2. Capacity-building programs to train therapists, teachers, and caregivers to design and deliver VR-based sessions.
3. Standardized protocols and outcome measures to allow comparability across studies are required.
4. Blended therapy models combine VR with face-to-face interventions to maximize transferability and clinical alignment.

### 4.3 Emerging opportunities

The use of new technologies to power virtual reality therapy has the potential to greatly enhance adaptation systems for providing personalization therapy which can be adjusted in real-time. These systems can alter the difficulty level and feedback techniques based on the performance and progress in therapy of every user. Wearable sensors and motion capture technologies enhance the accuracy of patient monitoring. Similarly, geolocation of tele-VR platforms significantly increases accessibility to therapists in remote or underserved geographical areas. Cross-domain applications offer very exciting potential for the intervention. These cross-domain applications are consistent with the broader literature on immersive technologies in experiential learning, post-stroke rehabilitation, upper-limb recovery, substance-use disorders, and addictive disorders (69–73). A virtual reality intervention for motor rehabilitation could also facilitate the development of cognitive and emotional skills, especially in those with comorbidities. VR modules designed to integrate social skills training along with components of motor activity within an autism spectrum disorder application may target behaviour and motor deficits in an individual group intervention. The combination of virtual reality, augmented reality and mixed reality technology together can offer certain therapeutic environments, allowing architects to design flexible hybrid solutions which integrate real-world and virtual-world elements. Through such Initiatives, the training may be continued to the various educational setups as well as the healthcare setups. By replicating real-world exercises in a controlled setting, our ability to participate in and appreciate natural events improves. Major research priority should be long-term ecological validation studies. We must investigate whether the neural skill acquired through virtual reality will withstand and transfer to ed, work, and community setting. A cost-benefit analysis will be important and need detailed analysis and done at scale. If these core research questions are answered, the virtual reality technology could undergo change from an experimental intervention to a proven treatment in clinical practice and classrooms.

### 4.4 Limitations of this review

While this systematic review and meta-analysis provides a comprehensive synthesis of the current evidence on VR interventions in ASD and PT, several methodological and practical limitations must be acknowledged when interpreting the findings.

#### 4.4.1 First, search and language restrictions

The literature search was restricted to three electronic databases (PubMed, Scopus, and Web of Science) and to articles published in English between 2020 and 2025. Although these databases provide broad coverage, relevant studies indexed exclusively in other databases (e.g., CINAHL, Embase, PsycINFO, Cochrane Central Register of Controlled Trials) or published in languages other than English may have been missed. Grey literature, including conference proceedings, dissertations, and unpublished studies, was excluded, which may contribute to publication bias by underrepresenting studies with null or negative findings.

#### 4.4.2 Second, database overlap and retrieval bias

As discussed in Section 2.2.2, Scopus and Web of Science are aggregator platforms that index content from multiple databases including MEDLINE, which is also directly accessible through PubMed. Although systematic deduplication removed 156 overlapping records, the reliance on three databases with partially overlapping coverage may not have captured the full breadth of relevant literature from specialized rehabilitation or technology-focused databases.

#### 4.4.3 Third, heterogeneity in study designs, populations, and outcome measures

The included studies varied considerably in terms of study design (RCTs, quasi-experimental, pre-post), participant characteristics (age groups ranging from children to elderly, clinical populations spanning ASD, stroke, Parkinson's disease, and musculoskeletal conditions), VR technology type (fully immersive, semi-immersive, non-immersive), intervention protocols (duration, frequency, session length), and outcome measurement instruments. This heterogeneity, reflected in the moderate I^2^ value of 44.3%, limits the precision of pooled effect size estimates and the specificity of clinical recommendations.

#### 4.4.4 Fourth, small sample sizes

The median sample size across included primary studies was 45 participants, with individual studies ranging from 18 to 156. Small samples reduce statistical power to detect true effects, increase the risk of inflated effect size estimates, and limit the generalizability of findings. Formal power analyses were not uniformly reported across included studies.

#### 4.4.5 Fifth, absence of control groups

Approximately 17.4% of included studies lacked a control group, employing single-arm pre-post designs instead. While these studies were analyzed separately in subgroup analyses, the absence of controlled comparisons increases the risk of confounding by natural recovery, practice effects, and regression to the mean.

#### 4.4.6 Sixth, publication bias

Funnel plot asymmetry and a statistically significant Egger's regression test (*p* = 0.0031) suggest the presence of small-study effects or publication bias. Studies with smaller samples and larger effect sizes appear to be overrepresented, which may inflate the pooled effect estimate. The exclusion of grey literature may further contribute to this bias.

#### 4.4.7 Seventh, limited long-term follow-up

Few included studies assessed the durability of VR intervention effects beyond the immediate post-intervention period. The absence of systematic long-term follow-up data (e.g., 3-month, 6-month, or 12-month assessments) prevents conclusions about whether observed therapeutic gains are sustained over time.

#### 4.4.8 Eighth, inclusion of secondary syntheses

Although secondary syntheses were excluded from quantitative computations, their inclusion in the qualitative discussion may introduce interpretive complexity. The cross-reference verification in Section 2.3 mitigates double-counting risk, but indirect overlap through shared underlying populations cannot be entirely excluded.

#### 4.4.9 Ninth, quality assessment tool

The 4-point quality scale used in this review is less granular than established tools such as the Cochrane Risk of Bias 2 (RoB 2) tool for RCTs or the Newcastle-Ottawa Scale for observational studies. Future reviews should employ domain-specific risk-of-bias instruments for more detailed appraisals of internal validity.

Despite these limitations, the consistent direction of effects across diverse study designs, populations, and VR technologies provides convergent evidence supporting the therapeutic utility of VR interventions in both ASD and PT domains. Future research addressing these limitations through larger multisite RCTs, standardized outcome measures, extended follow-up periods, and broader database coverage will strengthen the evidence base and facilitate the development of clinical guidelines.

## 5 Conclusion

Based on a systematic review and meta-analysis of trials, virtual reality is an effective adjunctive therapy for autism spectrum disorder (ASD) and physical therapy (PT). Through studies done between 2020 and 2025 of 23 trials, it has been found in the study review that VR consistently improves social communication, emotion recognition, and daily living skills in ASD while is also an effective tool for PT. It has been found through the study that VR improves balance and gait and upper limb function. The analysis showed that on average, included studies reported a moderate-to-large therapeutic benefit (Hedges' *g* = 0.66). The analyses suggested that most immersive systems (vs. any-control systems), as well as longer interventions (e.g., >2 h) groups, had beneficial effects. The value of VR lies not only in its clinical effectiveness but also in its capacity to increase motivation, engagement, and adherence to therapy, factors that are often critical for long-term progress in both neurodevelopmental and rehabilitation contexts. Nonetheless, challenges persist, including high equipment costs, limited therapist training, small sample sizes, and inconsistent methodological quality. Addressing these barriers through cost-effective VR solutions, capacity-building initiatives, standardized protocols, and blended therapeutic models is essential for broader clinical adoption. Looking ahead, VR may become a valuable adjunctive component of modern therapeutic practice, contingent on addressing current barriers including small sample sizes, methodological heterogeneity, limited long-term data, and publication bias. Emerging opportunities include AI-driven adaptive VR systems, tele-VR platforms for remote access, and hybrid VR/AR environments that seamlessly integrate digital and real-world contexts. Future research should focus on long-term follow-up, ecological validation of skills, and large-scale randomized trials to establish robust clinical guidelines. In conclusion, VR demonstrates a strong potential to transform therapeutic practice by offering immersive, engaging, and scalable interventions. With continued refinement and integration into clinical workflows, VR has the capacity to transition from experimental innovation to a promising evidence-informed tool, pending further large-scale trials that supports diverse populations across cognitive, behavioral, and physical domains.

## Data Availability

The original contributions presented in the study are included in the article/Supplementary Material, further inquiries can be directed to the corresponding author/s.
