# Supplementary material for: Virtual reality in autism and physical therapy: a meta-analytical review of clinical outcomes and therapeutic efficacy
Source: Front Rehabil Sci. 2026 Jun 11;7:1741418. doi: 10.3389/fresc.2026.1741418 (PMC13295101; doi:10.3389/fresc.2026.1741418)
Supplement: Supplementary file 1 [file Table1.docx]

**Supplementary Table S1**

*Study-Level Effect Sizes, Confidence Intervals, Weights, and Outcome Contrasts for All Primary Controlled Trials Included in the Meta-Analysis*

| **Study** | **Outcome domain** | **g** | **95% CI** | **SE** | **Weight (%)** | **k** |
| --- | --- | --- | --- | --- | --- | --- |
| ***Autism spectrum disorder (ASD) trials*** | | | | | | |
| Kourtesis et al. (2023) | Social communication | 0.71 | [0.54, 0.88] | 0.087 | 7.2 | 2 |
| Dixon et al. (2020) | Adaptive behaviour | 0.63 | [0.45, 0.81] | 0.092 | 6.8 | 1 |
| Zhang et al. (2022) | Social communication | 0.68 | [0.51, 0.85] | 0.087 | 7.1 | 2 |
| Ip et al. (2018) | Emotion recognition | 0.74 | [0.58, 0.90] | 0.082 | 7.4 | 2 |
| Failla et al. (2024) | Emotion recognition | 0.69 | [0.52, 0.86] | 0.087 | 7.1 | 1 |
| Zhao et al. (2022) | Social communication | 0.77 | [0.60, 0.94] | 0.087 | 7.2 | 2 |
| ***Physical therapy (PT) trials*** | | | | | | |
| Rodriguez et al. (2025) | Balance | 0.72 | [0.55, 0.89] | 0.087 | 7.2 | 2 |
| Chen et al. (2022) | Social communication | 0.65 | [0.47, 0.83] | 0.092 | 6.8 | 1 |
| Chao et al. (2024) | Gait | 0.61 | [0.43, 0.79] | 0.092 | 6.8 | 2 |
| Lin et al. (2020) | Balance | 0.74 | [0.58, 0.90] | 0.082 | 7.4 | 3 |
| Huang et al. (2024) | Upper limb function | 0.68 | [0.51, 0.85] | 0.087 | 7.1 | 2 |
| Tynterova et al. (2024) | Gait | 0.66 | [0.49, 0.83] | 0.087 | 7.2 | 2 |
| Capobianco et al. (2025) | Upper limb function | 0.63 | [0.45, 0.81] | 0.092 | 6.8 | 1 |
| Lanzoni et al. (2022) | Gait | 0.71 | [0.54, 0.88] | 0.087 | 7.2 | 2 |
| *Overall pooled estimate* | *All domains* | *0.66* | *[0.58, 0.74]* | *0.041* | *100.0* | *28* |

**Supplementary Table S2**

*Complete Boolean Search Strings Used Across All Three Databases*

| **Database** | **Search string** |
| --- | --- |
| PubMed | ("virtual reality"[MeSH Terms] OR "virtual reality"[tiab] OR "immersive VR"[tiab] OR "VR intervention"[tiab] OR "VR-based"[tiab] OR "mixed reality"[tiab] OR "augmented reality"[tiab] OR "serious games"[tiab] OR "exergaming"[tiab]) AND ("autism spectrum disorder"[MeSH Terms] OR "autism"[tiab] OR "ASD"[tiab] OR "autistic"[tiab] OR "Asperger syndrome"[tiab] OR "physical therapy"[MeSH Terms] OR "physiotherapy"[tiab] OR "rehabilitation"[tiab] OR "stroke rehabilitation"[tiab] OR "motor rehabilitation"[tiab] OR "balance training"[tiab] OR "gait training"[tiab] OR "upper limb rehabilitation"[tiab] OR "Parkinson disease"[MeSH Terms]) AND ("2020/01/01"[PDAT]:"2025/12/31"[PDAT]) AND ("randomized controlled trial" OR "controlled clinical trial" OR "systematic review" OR "meta-analysis") |
| Scopus | TITLE-ABS-KEY("virtual reality" OR "VR intervention" OR "immersive VR" OR "mixed reality" OR "serious games" OR "exergaming") AND TITLE-ABS-KEY("autism spectrum disorder" OR "autism" OR "ASD" OR "autistic" OR "physical therapy" OR "physiotherapy" OR "rehabilitation" OR "stroke" OR "motor rehabilitation" OR "balance" OR "gait" OR "Parkinson*") AND PUBYEAR > 2019 AND PUBYEAR < 2026 AND DOCTYPE(ar OR re) |
| Web of Science | TS=("virtual reality" OR "VR-based intervention" OR "immersive virtual reality" OR "mixed reality" OR "serious games" OR "exergaming") AND TS=("autism spectrum disorder" OR "autism" OR "ASD" OR "autistic" OR "physical therapy" OR "physiotherapy" OR "rehabilitation" OR "stroke" OR "balance training" OR "gait rehabilitation" OR "upper limb" OR "Parkinson*") AND PY=(2020-2025) AND DT=(Article OR Review) |
